# Proteome-wide Mendelian randomization provides novel insights into the pathogenesis and druggable targets of osteoporosis

**DOI:** 10.3389/fmed.2024.1426261

**Published:** 2024-10-25

**Authors:** Jingchuan Yan, Ying Huai, Qi Liang, Li Lin, Bo Liao

**Affiliations:** Department of Orthopedics, Tangdu Hospital, Fourth Military Medical University, Xi'an, China

**Keywords:** Mendelian randomization, osteoporosis, bone mineral density, proteomic, colocalization analysis, druggability evaluation

## Abstract

**Background:**

With the aging population, the prevalence and impact of osteoporosis are expected to rise, and existing anti-osteoporosis agents have limitations due to adverse events. This study aims to discover novel drug targets for osteoporosis.

**Methods:**

The protein data were obtained from the latest proteome-wide association studies (PWAS) including 54, 219 participants. The osteoporosis data were extracted from a GWAS meta-analysis, characterized by heel bone mineral density (HBMD) comprising 426,824 individuals. Mendelian randomization (MR) was the primary approach used to establish genetic causality between specific traits. Summary-data-based MR (SMR), colocalization analysis, heterogeneity test, and external validation were applied to ensure the findings were reliable. The underlying mechanisms behind these causal associations were investigated by additional analyses. Finally, the druggability of the identified proteins was assessed.

**Results:**

After Bonferroni correction, a total of 84 proteins were found to have a genetic association with osteoporosis. With strong colocalization evidence, proteins such as ACHE, HS6ST1, LRIG1, and LRRC37A2 were found to negatively influence HBMD, whereas CELSR2, CPE, FN1, FOXO1, and FSHB exhibited a positive association with HBMD. No significant heterogeneity was found. Additionally, CELSR2, FN1, FSHB, HS6ST1, LRIG1, and LRRC37A2 were replicated in the external validation. The effect of FSHB on HBMD was more pronounced in females compared to males. Interestingly, ACHE, LRIG1, FN1, and FOXO1 were observed to partially act on HBMD through BMI. Phewas analysis indicated that CPE and FOXO1 did not have genetic associations with any phenotypes other than osteoporosis. FN1 was highlighted as the most significant protein by protein-protein interaction network analysis.

**Conclusion:**

In conclusion, this study offers valuable insights into the role of specific proteins in the development of osteoporosis, and underscores potential therapeutic targets. Future studies should emphasize exploring these causal relationships and elucidating their underlying mechanisms.

## 1 Introduction

Osteoporosis is a systemic skeletal disease characterized by a decrease in bone density and the weakening of bone tissue microarchitecture (1). Patients with increased bone fragility are highly susceptible to fractures, thereby severely impairing their life quality (1). Current estimates indicate that more than 10.2 million people aged 50 and older are living with osteoporosis in the United States, incurring an annual cost of ~$57 billion (2). With the aging global population, the incidence of osteoporosis-related morbidity and disability is predicted to increase, presenting a significant challenge to healthcare systems around the world.

Despite favoring bone homeostasis, current anti-osteoporosis agents have limitations due to various adverse events. Prolonged use of bisphosphonates and denosumab is linked to a higher risk of atypical femoral fractures and osteonecrosis of the jaw (3). In addition, discontinuation of denosumab can result in the rapid onset of clusters of vertebral fractures, especially in patients with existing vertebral deformities (4, 5). Estrogen supplementation is associated with an increased risk of developing non-skeletal diseases, including breast cancer and cardiovascular conditions (5). The use of anabolic agents has raised alarms regarding the potential risk of osteosarcoma (5). Additionally, various factors such as contraindications, patient adherence, transition programs, and the occurrence of therapeutic plateaus can influence the effectiveness of osteoporosis therapies (3). Therefore, it is imperative to focus on developing new and safe therapeutic targets to address these issues.

Proteins are essential in numerous biological functions, acting as key markers for diseases and potential targets for drug development (6). Advancements in high-throughput genomic and proteomic techniques have facilitated large-scale genome-wide association studies (GWAS) to identify genetic factors influencing various plasma proteins, unveiling novel insights into the molecular mechanisms of diseases (7, 8). Mendelian Randomization (MR) analysis, which uses genetic variants as instrumental variables, has become an invaluable method for reducing the influence of confounders and reversing causality often seen in observational studies, thus proving to be a robust approach for establishing causal relationships between exposures and outcomes (9). By leveraging protein quantitative trait loci (pQTLs) identified through proteome-wide association studies (PWAS), proteome-wide MR can assess the causality between plasma proteins and disease outcomes, aiding in the identification of targets suitable for drug development (9). Proteome-wide MR studies have been conducted in diverse diseases, including multiple sclerosis (10), colorectal cancer (11), type 2 diabetes (12), and inflammatory bowel disease (13). Although previous MR studies have examined the links between diseases and osteoporosis (14–17), a comprehensive investigation of druggable targets for osteoporosis using proteome-wide MR has yet to be extensively pursued.

In this study, the pQTLs data was extracted from a comprehensive and updated large-scale PWAS. Multiple methods were employed to validate the robustness of the MR findings and to delve into the mechanisms potentially driving the identified causal relationships. The discovery of proteins with a causal link to osteoporosis could enhance our understanding of genetic architecture and accelerate the development of drug targets for this disease.

## 2 Materials and methods

### 2.1 Study design and data available

**Figure 1:**
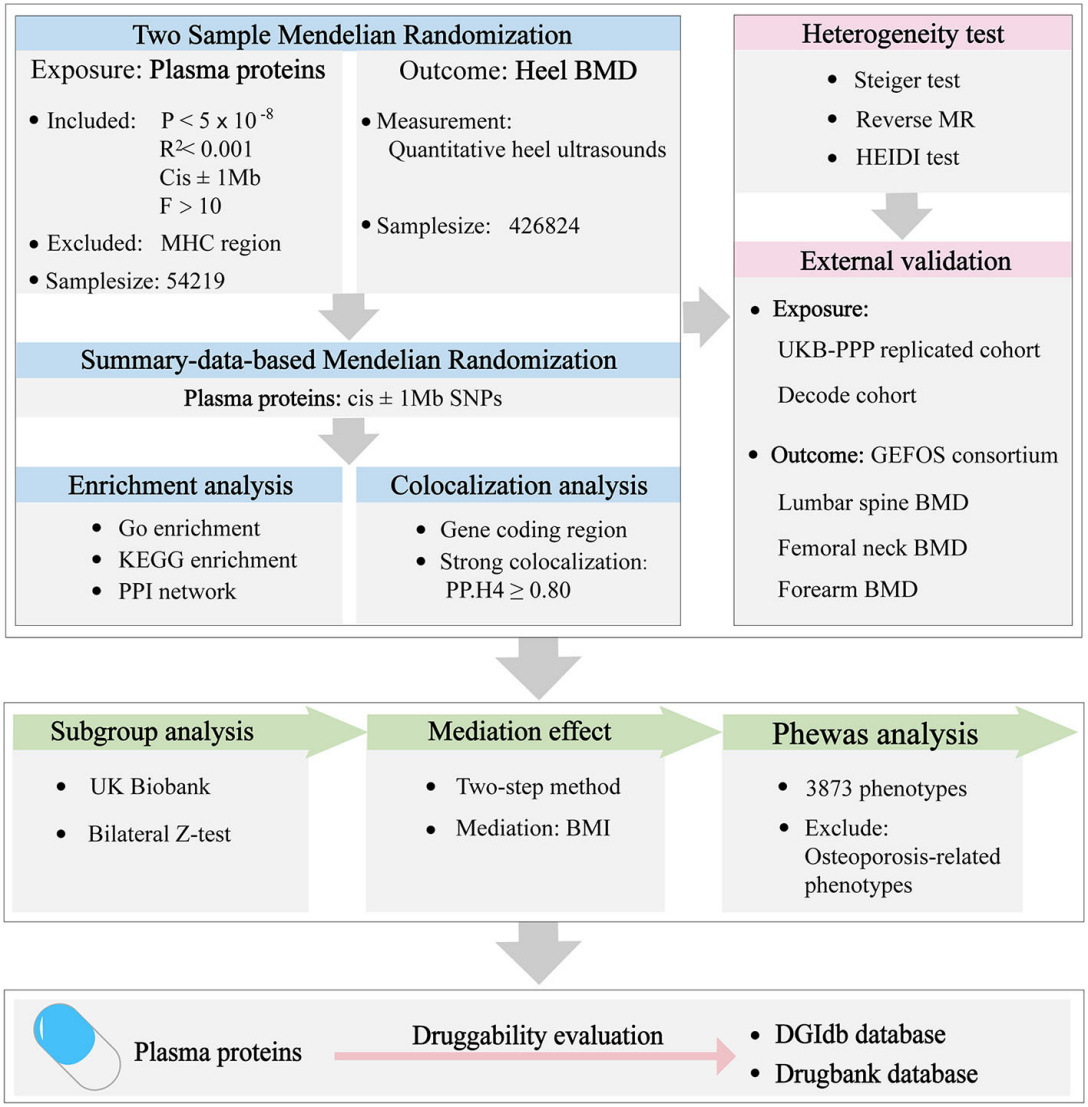
The study design of this Mendelian randomization analysis.

A proteome-wide Mendelian randomization was conducted to investigate the causal associations between the plasma proteins and osteoporosis. All the summary data utilized were sourced from publicly available, previously published large-scale GWAS studies. All the databases used in this paper are shown in Supplementary Table 1. Details about these databases are described in the original article. As a result, this research did not necessitate additional ethical approval or informed consent. The overview of the study design is shown in Figure 1.

### 2.2 Mendelian randomization

The summary pQTLs data of plasma proteins were obtained from the discovery cohort of the Pharma Proteomics Project in the UK Biobank (UKB-PPP) (http://ukb-ppp.gwas.eu) (7). This project offers a recent and detailed analysis of the genetic framework underlying the proteomic profiles of plasma in a cohort of 54,219 participants. Comprehensive genetic associations between pQTLs and 2,923 proteins were identified by the Olink platform. Additionally, the summary data for osteoporosis was derived from the largest GWAS study on bone mineral density assessed using quantitative heel ultrasounds (HBMD), encompassing 426,824 European individuals (https://gwas.mrcieu.ac.uk/) (18).

The genetic instruments of plasma proteins were considered valid in this study after they met the following criteria: first, single nucleotide polymorphisms (SNPs) were strongly associated with the plasma proteins (*P* < 5 × 10^−8^); second, SNPs were clumped based on 1,000 Genomes European panel (*R*^2^ < 0.001) to avoid the linkage disequilibrium; third, cis SNPs within 1 Mb from gene encoding the protein were included; Fourth, SNPs with *F*-statistics >10 were included to mitigate the risk of weak instrument bias; Fifth, SNPs located in major histocompatibility complex (MHC) region (chr 6, 26–34 Mb) were excluded to avoid confounding factors. The matched human genome build served as the reference for completing any missing information in the dataset. Eventually, 1,869 cis-pQTLs of plasma proteins were included in MR analysis (Supplementary Table 2). The Wald Ratio method was used to evaluate the cis-pQTLs with one SNP, whereas the Inverse Variance Weighted (IVW) method was employed for cis-pQTLs with two or more SNPs. To avoid false positive errors, *P* < 2.675 × 10^−5^ (0.05/1,869) was considered a statistically significant result after the Bonferroni correction. All analyses were performed by package “TwoSampleMR” in R (version 4.1.2).

We validated the reliability of the aforementioned results by the Summary-data-based MR (SMR) method (19, 20). Compared to conventional MR analysis, SMR analysis infers causality based on the top associated cis-QTL (19). When data for exposure and outcome come from two separate datasets with large sample sizes, SMR achieves significantly enhanced statistical power (19). For the analysis, genetic variants located within 1,000 kilobase (kb) on either side of the coding sequence (referred to as cis-QTLs) that displayed strong associations with gene expression with a significance threshold of *P* < 5.0 × 10^−8^, were included. The threshold for statistical significance was adjusted using Bonferroni correction to *P* = 5.8 × 10^−4^ (0.05/85), ensuring that the risk of Type I errors was minimized in the context of multiple testing. All SMR analyses were conducted using the software “SMR v1.3.1” (19).

### 2.3 Colocalization analysis

Colocalization analysis is an effective method to confirm causation is directly linked to a shared genetic variant rather than due to linkage disequilibrium (11). The analysis included five distinct hypotheses: H0, no causal variant for either exposure or outcome; H1/H2, each positing one causal variant for either the exposure or outcome alone; H3, two different causal variants for exposure and outcome; H4, proposing a shared causal variant affecting both traits. SNPs within 1 Mb from gene coding the protein were extracted for colocalization analysis in plasma proteins (7) and HBMD data (18). A posterior probability for H4 (PP.H4) exceeding 0.80 was deemed to provide strong evidence of colocalization (11). The “coloc” package was utilized for this analysis with default settings (prior probability an SNP is associated with exposure, *P*1 = 1 × 10^−4^; prior probability an SNP is associated with outcome, *P*2 = 1 × 10^−4^; prior probability an SNP is associated with both exposure and outcome, *P*12 = 1 × 10^−5^) and the package “LocusCompareR” package was employed to visualize these results (11).

### 2.4 Heterogeneity test and external validation

Steiger directionality test was performed to detect the correct causal direction (10). To further confirm the absence of reverse causation between plasma proteins and HBMD, reverse MR analyses were conducted. In addition to colocalization analysis, the heterogeneity in dependent instruments (HEIDI) test was employed as another means of verifying causation directly attributable to a shared genetic variant rather than being a result of linkage disequilibrium based on SMR analysis (19). The results were considered statistically significant at *P* < 0.05.

External validation was used to validate the robustness of causal associations between plasma proteins and HBMD. Firstly, the cis-pQTL from the UKB-PPP replicated cohort (http://ukb-ppp.gwas.eu) (7) were analyzed to determine if the initial findings could be corroborated, thus enhancing the credibility of the observed associations. Secondly, as described previously (10), SNPs were retrieved from GWAS datasets related to the corresponding proteins, made available by the Decode genetics consortium (https://www.Decode.com/summarydata/) (8) to further increase the overall persuasiveness of the results. Thirdly, to replicate and validate the causal findings of identified proteins on HBMD, the summary data of osteoporosis-related traits measured by Dual-energy X-ray Absorptiometry (DXA) were extracted from the GEnetic Factors for Osteoporosis (GEFOS) Consortium (http://www.gefos.org/) (21), which included lumbar spine (LS), femoral neck (FN) and forearm (FM) BMD, comprising the 33,297, 32,735, and 8,143 European participants. All analyses were performed by package “TwoSampleMR” in R (version 4.1.2) and *P* < 0.05 was regarded as a significant result.

### 2.5 Additional analysis

Given the well-documented differences in osteoporosis pathogenesis between men and women, a sex-stratified subgroup analysis was conducted to explore the potential sex-specific effects of the identified proteins on BMD. The summary data of gender-specific HBMD were obtained from the UK Biobank (https://www.nealelab.is/uk-biobank) (22). The difference between groups was evaluated by bilateral *Z*-test and *P* < 0.05 was considered significant.

To explore the potential role of body mass index (BMI) in the progression of osteoporosis, a two-step MR analysis (23) was conducted to investigate the mediating effect of BMI between identified proteins and HBMD. The first step was applied to identify proteins that are associated with BMI (MRC-IEU consortium; 461,460 European participants) (https://gwas.mrcieu.ac.uk/). The second step was employed to explore the association between BMI and HBMD outcomes. The indirect effect mediated by BMI was calculated using the coefficient product method, and the corresponding standard error was calculated using the Delta method (23). *P* < 0.05 was considered as significant.

Phewas, a genotype-to-phenotype approach, was conducted using summary statistics to examine systematically the associations of tag SNPs for proteins with a wide range of diseases and related traits, which helped to discover the potential pleiotropy (24). The criteria for filtering the data and categorizing the numerous phenotypes followed the methodology outlined in a previous study (24). In brief, phenotypic data from the European population within the UK Biobank were utilized for this analysis. Continuous phenotypes were categorized based on recommendations from the UK Biobank, while binary outcomes were organized according to relevant chapters of the ICD-9/10 classification system, ensuring an etiologically coherent grouping. To maintain adequate statistical power, binary phenotypes with fewer than 100 cases and continuous phenotypes with sample sizes below 10,000 were excluded. Additionally, certain phenotypes not directly related to health status, such as family history, household, and sociodemographic factors, were also removed from consideration. Finally, 3,873 phenotypes were included to scan the phenotypes associated with identified proteins using packages “Ieugwasr” and “TwoSampleMR.” *P* < 0.05/3,873/Number of proteins was considered significant after Bonferroni correction.

### 2.6 Enrichment analysis and protein-protein interaction network

Gene enrichment analysis was conducted to investigate the characteristics and biological relevance of putative proteins. This analysis encompassed Gene Ontology (GO) enrichment, covering aspects such as biological processes (BP), molecular functions (MF), and cellular components (CC), as well as Kyoto Encyclopedia of Genes and Genomes (KEGG) enrichment, which could shed light on the pertinent metabolic pathways (25). The packages “org.Hs.eg.db” and “enrichplot” were used to perform enrichment analysis and visualize the results. To mitigate the risk of false positive findings, the *P*-value was adjusted using the false discovery rate (FDR) method.

To gain insights into the interactions among the identified proteins, a Protein-Protein Interaction (PPI) network was constructed using the STRING database (https://cn.string-db.org/). The default parameters were adopted and the minimum needed interaction score was 0.4. The software “Cytoscape” (26) was employed to visualize these PPI networks and to perform calculations for each node's score, facilitating the identification of the central or hub protein within the network.

### 2.7 Druggability evaluation

The DGIdb database (https://dgidb.genome.wustl.edu/) was utilized to determine if the identified proteins belonged to the category of druggable genomes, which are theoretically capable of being targeted by therapeutic compounds. To expand on this, the Drugbank database (https://go.drugbank.com/) was searched to find existing drugs that act on these potentially causal proteins. In this context, only approved drugs with clear pharmacological action were considered to be included due to clearly defined medicinal effects.

## 3 Results

### 3.1 Mendelian randomization and colocalization analysis

**Figure 2:**
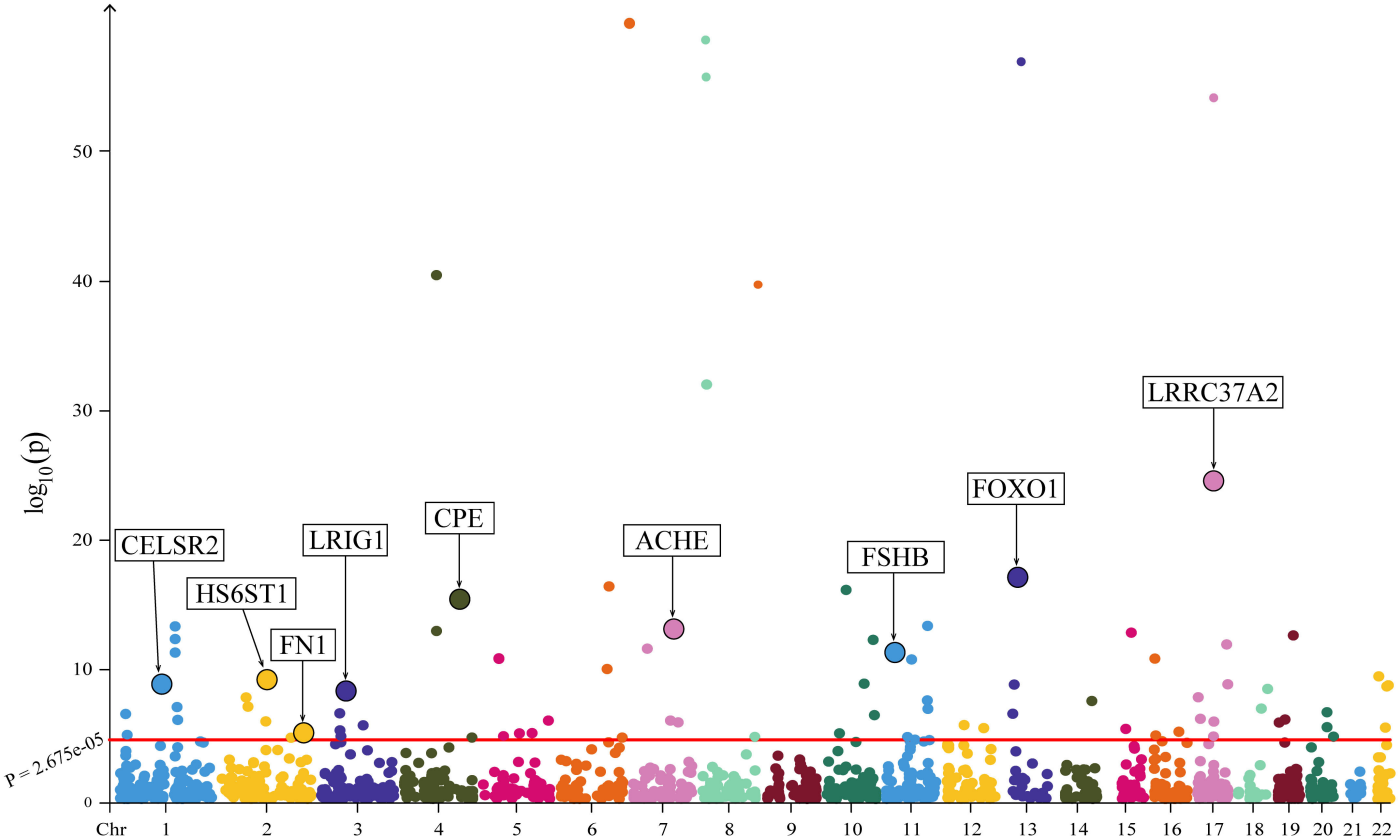
The Manhattan plot illustrating the associations between plasma proteins and HBMD. The *X*-axis represents chromosomal position; the *Y*-axis represents negative log_10_-transformed *p*-values; nine proteins demonstrating strong colocalization evidence are highlighted.

All the MR results of proteins are shown in Figure 2. After Bonferroni correction, 85 plasma proteins showed significant associations (*P* < 2.675 × 10^−5^) with osteoporosis, among which 38 proteins were negatively associated with HBMD, whereas 47 proteins were positively associated with HBMD. To further validate these findings, the plasma proteins were subjected to SMR analysis, and only GP1BB was eliminated after Bonferroni correction. Notably, the directionality of all SMR results was consistent with MR analysis (Supplementary Table 3).

Colocalization analysis was performed to explore the shared genetic basis between the remaining 84 plasma proteins and HBMD, as indicated in Supplementary Table 4. Among them, nine proteins demonstrated strong colocalization evidence (PP.H4 ≥ 0.80), including CPE (PP.H4 = 0.999), LRIG1 (PP.H4 = 0.999), FOXO1 (PP.H4 = 0.997), CELSR2 (PP.H4 = 0.985), HS6ST1 (PP.H4 = 0.980), ACHE (PP.H4 = 0.971), FN1 (PP.H4 = 0.939), FSHB (PP.H4 = 0.890) and LRRC37A2 (PP.H4 = 0.807). Table 1 depicts the main results for the identified nine proteins. 5 plasma proteins displayed weak colocalization evidence (0.50 ≤ PP.H4 < 0.80). In contrast, 68 plasma proteins did not show significant colocalization evidence (PP.H4 < 0.50). In addition, colocalization analysis could not be conducted for two plasma proteins due to the lack of matching SNPs with HBMD.

**Table 1:** MR results for nine plasma proteins supported by strong colocalization evidence.

**Tissue**	**Proteins^a^**	**UniProt ID**	**SNP**	**Effect allele**	**Beta (95% CI)**	***P*-value**	**PVE**	**F statistics**	**PP.H4**	**P1**	**P2**	**P3**
Plasma	ACHE	P22303	rs6976053	T	−0.04 (−0.06, −0.03)	6.18E−14	2.79%	951.71	0.971	< 0.001	0.27	< 0.001
Plasma	CELSR2	Q9HCU4	rs12740374	T	0.03 (0.02, 0.04)	2.88E−09	2.67%	909.01	0.985	< 0.001	0.67	0.04
Plasma	CPE	P16870	rs1550270	C	0.06 (0.05, 0.08)	1.27E−15	1.20%	408.93	0.999	< 0.001	0.44	0.01
Plasma	FN1	P02751	rs1250259	A	0.02 (0.01, 0.03)	9.94E−06	3.19%	1,118.85	0.937	< 0.001	0.68	0.67
Plasma	FOXO1	Q12778	rs17061453	T	0.14 (0.11, 0.18)	1.12E−17	0.05%	19.02	0.997	< 0.001	0.7	0.04
Plasma	FSHB	P01225	rs11031006	A	0.12 (0.09, 0.16)	4.2E−12	0.30%	100.55	0.891	< 0.001	0.39	< 0.001
Plasma	HS6ST1	O60243	rs6761320	T	−0.05 (−0.07, −0.04)	4.89E−10	1.13%	387.21	0.98	< 0.001	0.8	< 0.001
Plasma	LRIG1	Q96JA1	rs2306272	C	−0.01 (−0.02, −0.01)	1.02E−08	13.25%	5,171.74	0.999	< 0.001	0.62	0.63
Plasma	LRRC37A2	A6NM11	rs62057151	T	−0.02 (−0.02, −0.02)	3.74E−25	16.09%	6,477.92	0.807	< 0.001	0.43	< 0.001

PVE, proportion of variance explained; P1, Steiger test; P2, Reverse MR; P3, HEIDI test.

^a^All the plasma protein information was referenced to Sun et al. (7).

### 3.2 Heterogeneity test and external validation

The heterogeneity test was primarily directed at plasma proteins with strong evidence of colocalization. The *P*-values for these proteins, as determined by the Steiger test, were all below 0.05, suggesting the correct direction of causality. Moreover, reverse causal analysis showed that HBMD did not have a causal impact on these plasma proteins. These findings from both approaches confirmed the absence of a reverse causal relationship between these plasma proteins and HBMD, as documented in Table 1.

In the primary analysis, the results from the UKB-PPP discovery cohort demonstrated that ACHE (beta = −0.04; 95% CI = −0.06 to −0.03), HS6ST1 (beta = −0.05; 95% CI = −0.07 to −0.04), LRIG1 (beta = −0.01; 95% CI = −0.02 to −0.01) and LRRC37A2 (beta = −0.02;95% CI = −0.02 to −0.02) were negatively associated with HBMD, whereas CELSR2 (beta = 0.03; 95% CI = 0.02–0.04), CPE (beta = 0.06; 95% CI = 0.05–0.08), FN1 (beta = 0.02; 95% CI = 0.01–0.03), FOXO1 (beta = 0.14; 95% CI = 0.11–0.18) and FSHB (beta = 0.12; 95% CI = 0.09–0.16) were positively associated with HBMD (Table 1). The UKB-PPP replicated cohort yielded consistent results, as shown in Supplementary Tables 5, 7.

To further confirm the causal relationships identified earlier, SNPs linked to the nine specified proteins were sourced from the Decode study. However, data on the two proteins, CELSR2 and FOXO1, were absent in the cohort. Additionally, four SNPs weakly associated (*P* > 1 × 10^−5^) with corresponding proteins (ACHE, CPE, FSHB, LRRC37A2) were omitted for not meeting the relevance criteria in MR analysis. Consequently, only three proteins from the Decode study were incorporated into the MR analysis. Consistent with the aforementioned results, HS6ST1 (beta = −0.08; 95% CI = −0.1 to −0.05) and LRIG1 (beta = −0.02; 95% CI = −0.02 to −0.01) were negatively associated with HBMD, but FN1 (beta = 0.05; 95% CI = 0.03–0.08) was positively associated with HBMD (Supplementary Tables 6, 7).

Among the summary data from the GEFOs Consortium, CELSR2 was found to have a positive impact on LS BMD (beta = 0.06; 95% CI = 0.02–0.11). LRRC37A2 was observed to negatively influence LS TBM (beta = −0.04; 95% CI = −0.06 to −0.02), while HS6ST1 had a negative causal effect on FN BMD (beta = −0.08; 95% CI = −0.15 to −0.01) and LS BMD (beta = −0.02; 95% CI = −0.03 to −0.004); Interestingly, FSHB significantly affected all these traits, including FM BMD (beta = 0.44; 95% CI = 0.14–0.75), FN BMD (beta = 0.15; 95% CI = 0.01–0.30) and LS BMD (beta = 0.20; 95% CI = 0.04–0.37). All these results were consistent with the previous findings (Supplementary Table 7).

### 3.3 Additional analysis

**Figure 3:**
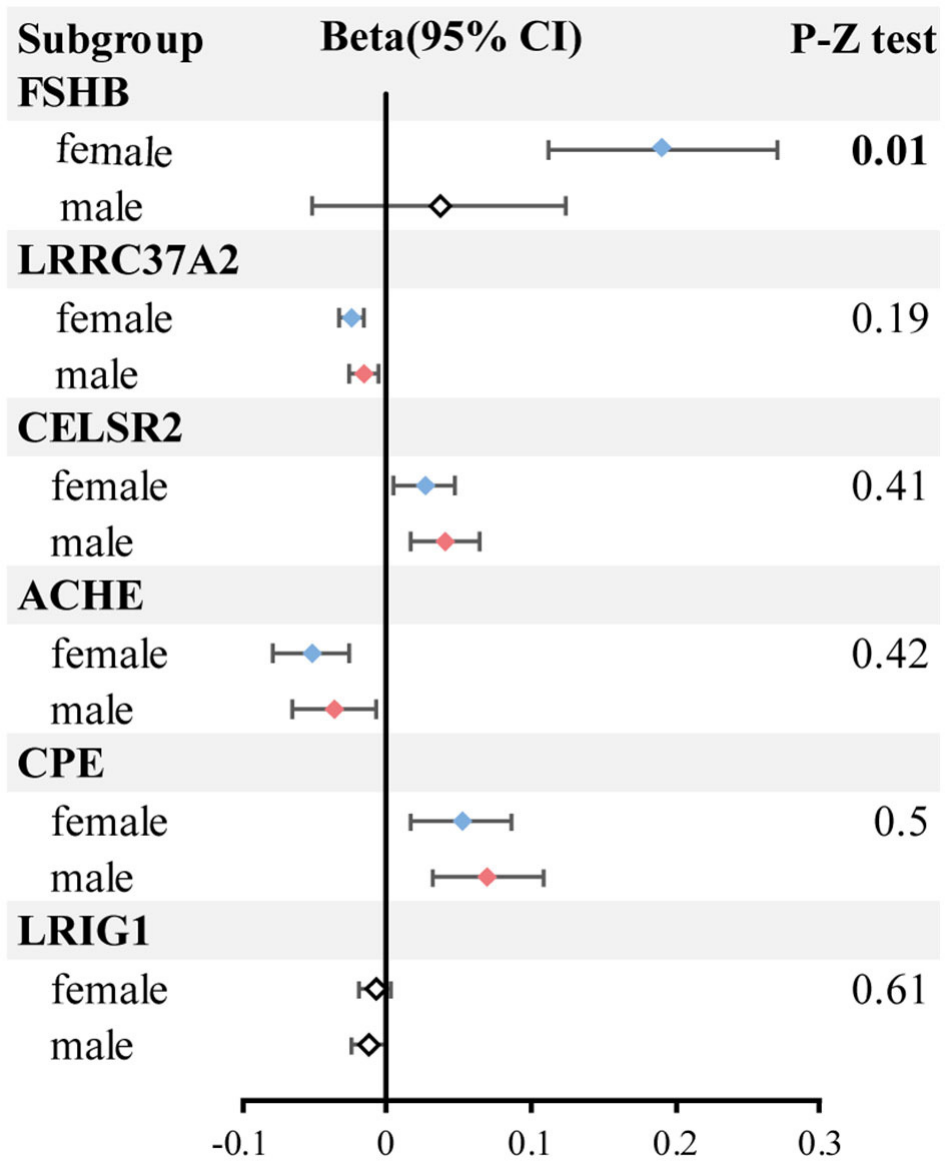
The forest plot of subgroup analysis. The blue represents the female group with significant results; the red represents the male group with significant results; the white box represents no significant results.

In subgroup analysis, HS6ST1 was not retrievable from the three types of outcome summary data (both-sexes, female and male HBMD). Two proteins (FN1 and FOXO1) were removed after harmonization between exposure and outcome due to ambiguous alleles. The remaining six proteins (ACHE, CELSR2, CPE, FSHB, LRIG1, LRRC37A2) consistently demonstrated causal effects on HBMD for both sexes, aligning with the previous results. However, LRIG1 did not have a causal effect on female HBMD (*P* = 0.167), while FSHB (*P* = 0.404) and LRIG1 (*P* = 0.051) were not associated with male HBMD. Bilateral *Z*-test results indicated that only FSHB had a significantly more positive effect on female HBMD (beta = 0.20; 95% CI = 0.11–0.23) compared to male HBMD (beta = 0.04; 95% CI = −0.05 to 0.08; *z*-test *P*-value = 0.011; Figure 3; Supplementary Table 8).

**Figure 4:**
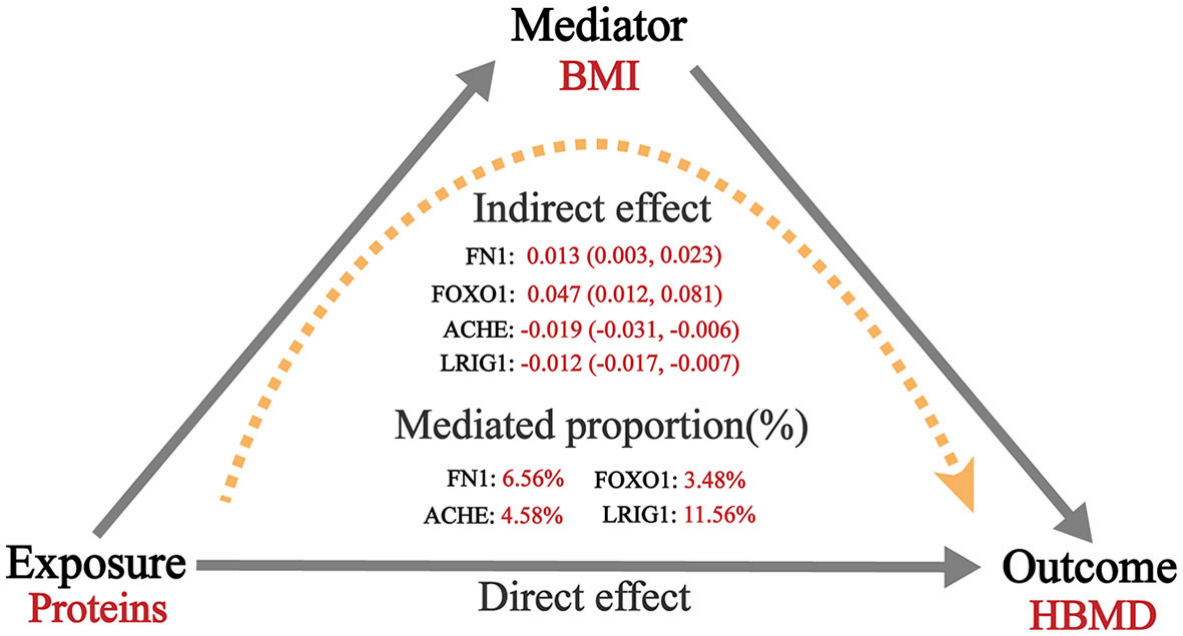
Mediation analysis of the effect of plasma proteins on HBMD.

MR analysis exploring mediation effects revealed that the causal relationships between four proteins and BMD were partially mediated by BMI. In the first step, ACHE (beta = −0.019; 95% CI = −0.031 to −0.006) and LRIG1 (beta = −0.012; 95% CI = −0.017 to −0.007) were negatively associated with BMI, whereas FN1 (beta = 0.013; 95% CI = 0.003–0.023) and FOXO1 (beta = 0.047; 95% CI = 0.012–0.081) were positively associated with BMI. In the second step, BMI was shown to be positive with HBMD (beta = 0.11; 95% CI = 0.08–0.14). Therefore, the indirect effect of ACHE, LRIG1, FN1 and FOXO1 on HBMD via BMI were 4.58% (beta = −0.002; 95% CI = −0.004 to −0.007), 11.56% (beta = −0.001; 95% CI = −0.002 to −0.007), 6.56% (beta = 0.0014; 95% CI = 0.0003–0.003) and 3.48% (beta = 0.005; 95% CI = 0.0012–0.0093), respectively (Figure 4; Supplementary Table 9).

Phewas analysis was conducted to test for the presence of pleiotropy among the proteins identified. After adjusting for multiple comparisons using the Bonferroni method (*P* < 1.43 × 10^−6^), several significant associations were discovered: ACHE was associated with diastolic blood pressure, leg and whole body impedance, red blood cell distribution width, pulse rate, and standing height, indicating its broad influence on cardiovascular and physiological traits. CELSR2 showed associations with lipid profiles and circulatory disorders, highlighting its potential role in metabolic and cardiovascular health. FN1 was linked to systolic blood pressure, platelet crit, and standing height, suggesting its impact on vascular health and physical attributes. FSHB was predominantly associated with gynecologic and obstetric disorders, underscoring its relevance in reproductive health. HS6ST1 showed an exclusive association with standing height, suggesting a specific role in growth or skeletal development. LRIG1 was associated with a range of phenotypes including fat mass, leg impedance, systolic blood pressure, glycated hemoglobin, mean corpuscular hemoglobin, and red blood cell distribution width, indicating its involvement in metabolic health, cardiovascular function, and blood characteristics. LRRC37A2 was mainly related to physical measures, mental health, blood count, and biochemistry, suggesting a broad influence on general health and wellbeing. However, CPE and FOXO1 did not show associations with any phenotypes apart from BMD, highlighting their potentially specific roles in bone health. The detailed results of Phewas analysis are shown in Supplementary Tables 10–18.

### 3.4 Enrichment analysis and PPI

**Figure 5:**
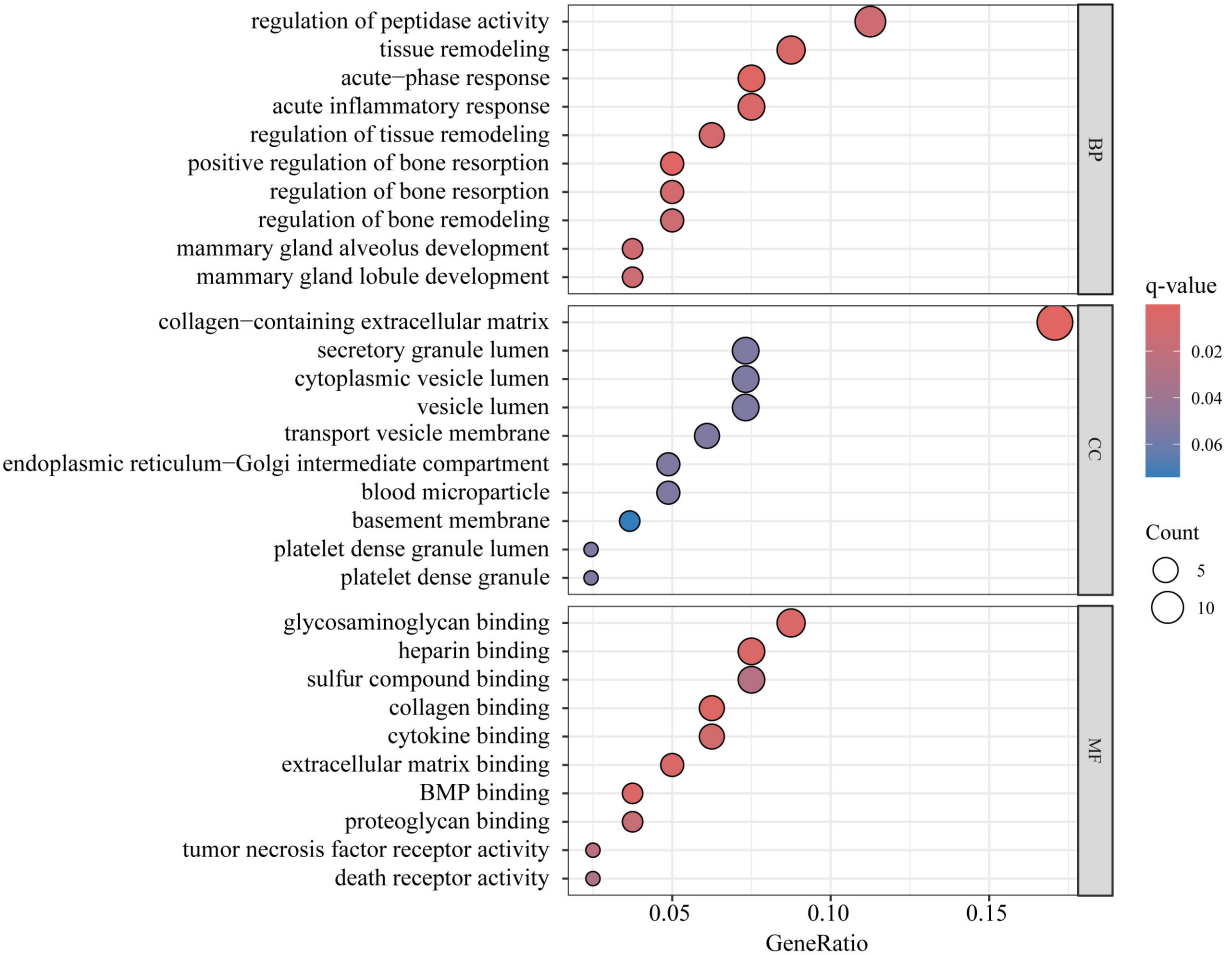
The Bubble diagram of GO enrichment analysis.

The proteins that showed positive results in both MR and SMR analysis were used for enrichment analysis. GO enrichment analysis identified 80 genes encoding protein, while KEGG enrichment analysis pinpointed 58 genes encoding protein. Following FDR correction, the top GO enrichment categories were regulation of peptidase activity within BP, collagen-containing extracellular matrix in MF, and glycosaminoglycan binding in Cellular Components CC, as illustrated in Figure 5 and detailed in Supplementary Table 19. The ECM-receptor interaction was identified as the sole significant metabolic pathway in the KEGG enrichment analysis (Supplementary Table 20).

**Figure 6:**
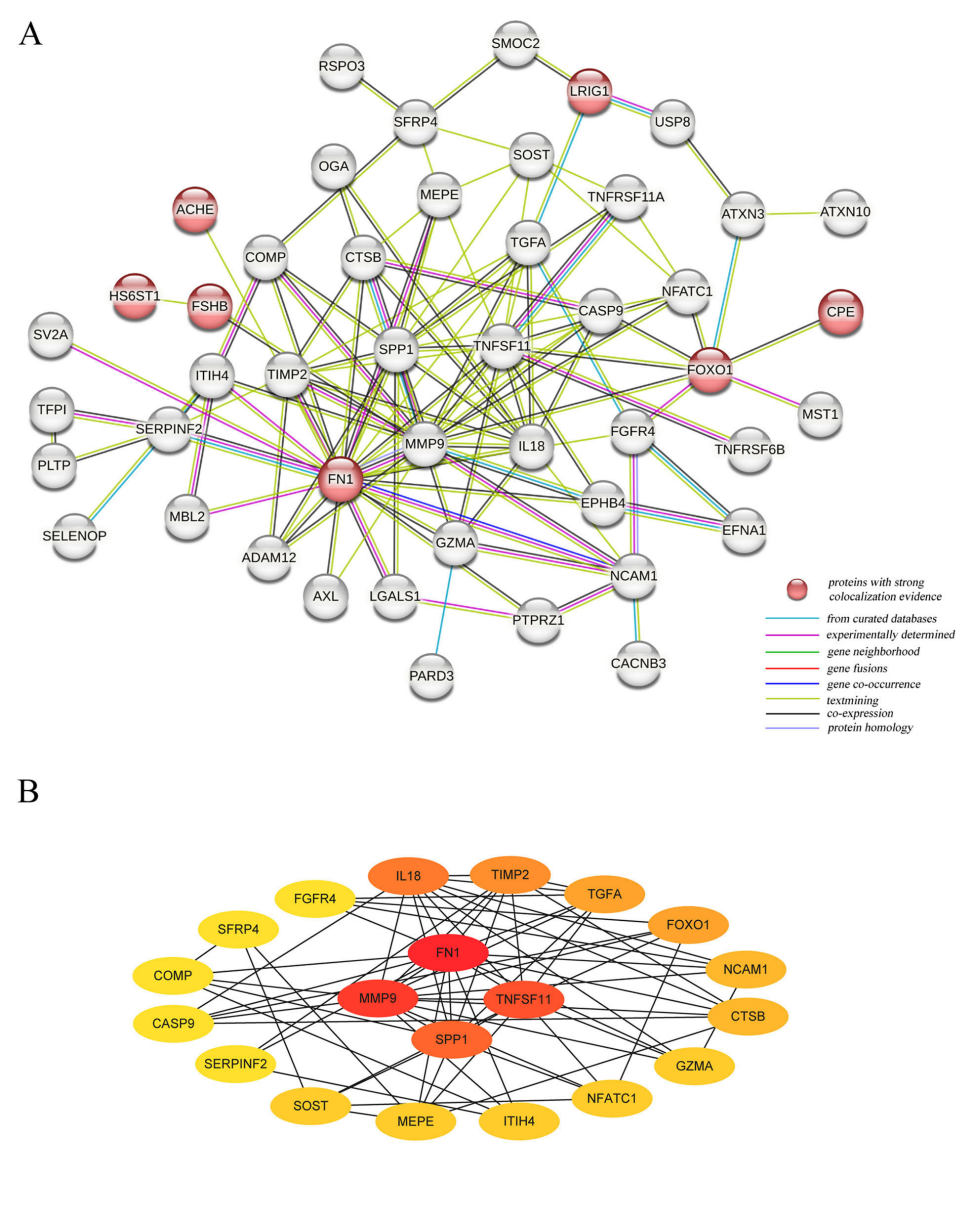
The Protein-Protein interaction network for identified plasma proteins. **(A)** The connections between 47 plasma proteins significantly associated with HBMD; **(B)** The 20 Hubba proteins based on the score per node.

The PPI network revealed connections among 47 of the 84 plasma proteins (Figure 6A). Notably, there were three significant interactions between proteins with strong colocalization evidence: HS6ST1 with FSHB, FOXO1 with CPE, and ACHE with FN1. Among these proteins, FN1 emerged as the most significant hub protein, highlighting its critical role in the molecular mechanisms driving osteoporosis (Figure 6B).

### 3.5 Druggable evaluation

We searched nine identified proteins with strong colocalization evidence as possible drug targets in drug databases. Five protein-encoding genes were identified as a part of the druggable genome: *ACHE, CELSR2, CPE, FN1, and FSHB*. Among them, ACHE and FN1 are already targeted by approved drugs, hinting at their potential therapeutic role in treating osteoporosis. Specifically, 22 drugs were identified as inhibitors of ACHE, while Pralidoxime and Pralidoxime chloride were found to activate ACHE. Additionally, Ocriplasmin was noted for its ability to cleave the protein FN1. More detailed information can be found in Supplementary Tables 21, 22.

## 4 Discussion

In this study, we performed proteome-wide Mendelian randomization to investigate the causal associations between 1,869 proteins and HBMD with the following findings. After applying Bonferroni correction, 84 proteins were identified as having a genetic causal relationship with osteoporosis. Notably, nine proteins demonstrated strong colocalization evidence. Among them, ACHE, HS6ST1, LRIG1, and LRRC37A2 were negatively associated with HBMD, while CELSR2, CPE, FN1, FOXO1 and FSHB were positively associated with HBMD. Our analysis did not reveal any reverse causal relationships. The HEIDI test provided additional support for the causal associations involving LRIG1 and FN1. Additionally, CELSR2, FN1, FSHB, HS6ST1, LRIG1, and LRRC37A2 were replicated in the external validation. Subgroup analyses indicated that FSHB exerted a more pronounced positive impact on HBMD in females than in males. Furthermore, it was discovered that ACHE, LRIG1, FN1, and FOXO1 influenced HBMD partially through BMI. Phewas analysis revealed no evidence of potential pleiotropy for CPE and FOXO1, suggesting that their associations with osteoporosis were specific and not influenced by other phenotypic traits. GO enrichment analysis highlighted significant biological functions, including the regulation of peptidase activity, the presence of a collagen-containing extracellular matrix, and glycosaminoglycan binding. Additionally, KEGG enrichment analysis identified the ECM-receptor interaction as the only significant metabolic pathway, further emphasizing the importance of extracellular matrix interactions in the disease. The PPI network demonstrated that FN1 was the most significant hub protein among 47 proteins. Importantly, the druggable evaluation revealed that ACHE and FN1 were targets of currently approved drugs, highlighting the importance of existing medications that could potentially be repurposed or serve as the basis for the development of new treatments for osteoporosis.

The present MR study provides strong additional evidence for the seven proteins and osteoporosis from a genetic perspective. For example, carboxypeptidase E (CPE), known for its role in processing prohormones and proneuropeptides, has been identified as having a significant association with diabetes, hyperproinsulinemia, and reduced BMD (27, 28). Moreover, bioinformatics analysis identified *CPE* as a pivotal hub gene among differentially expressed mRNAs linked to osteoporosis (29). This finding is supported by basic research indicating that mice lacking *CPE* display characteristics such as decreased BMD, increased bone turnover, increased expression of RANKL (a key regulator of bone remodeling), an absence of mature cocaine- and amphetamine-regulated transcript (CART) and so on (28). This positive effect of CPE on BMD was further corroborated by an independent MR study (30). Acetylcholinesterase (ACHE), a classic cholinergic hydrolase has also been implicated in bone physiology (31). Recently, acetylcholinesterase inhibitors (AChEIs), primarily Donepezil and Rivastigmine, which are drugs used to treat Alzheimer's disease (AD), have been shown to have a beneficial effect on bone health (32). These drugs enhance bone protection by activating acetylcholine receptors and inhibiting the function of osteoclasts, the cells responsible for bone resorption (32). This inhibition occurs through the modulation of signaling pathways such as MAPK and NFATc1 (32), which are crucial for the differentiation and activity of osteoclasts. Furthermore, a cohort study revealed that the use of AChEIs is associated with a clinically significant decrease in the risk of fractures in men with dementia, even after adjusting for other confounding factors (33), which remained consistent with another multicenter study (34). Forkhead box O1 (FOXO1) is a transcription factor that is pivotal in bone biology, particularly in promoting bone formation. It exerts its effects by enhancing the proliferation of osteoblasts and by maintaining the redox balance within these cells (35, 36). Besides, baicalein, a naturally occurring compound, has been shown to upregulate FOXO1 and thus enhance the expression of bone turnover markers and extracellular matrix mineralization, thereby ameliorating glucocorticoid-induced osteoporosis in cellular models, highlighting the potential therapeutic benefits of baicalein in bone health management (37, 38). Unfortunately, limitations in current proteomics databases prevented us from conducting external validation for these three proteins. However, the findings of present MR study are in strong agreement with previous research, which underscores the importance of these proteins in regulating bone metabolism and addressing osteoporosis.

Even though two proteins (HS6ST1 and CELSR2) have been reported to be associated with BMD (18), there has been little basic research focusing on these associations. HS6ST1 plays a vital role in the biosynthesis of heparan sulfate, a critical component of both the extracellular matrix (ECM) and cell surface (39). Our enrichment analysis highlighted the role of ECM in osteoporosis, suggesting a link between HS6ST1 and the disease. Furthermore, HS6ST1 has been shown to influence the Wnt signaling pathway, both directly and indirectly (40). The Wnt/β-catenin signaling is known to control the bone remodeling process and plays a role in the development of osteoporosis (41). CELSR2, traditionally linked with neuronal development, has also been found to play a role in non-neurological diseases and could be associated with osteoporosis through its involvement in lipid metabolism, endoplasmic reticulum stress, and reactive oxygen species (ROS) (42–46). It is worth mentioning that the silencing of CELSR2 could inhibit the Wnt signaling in Schwann cells (47). Fibronectin 1 (FN1), a member of the glycoprotein family of ligands has been implicated to play an important role in cell migration, adhesion, and cytoskeleton organization (41). Interestingly, basic research has revealed that an increase in FN1 expression can aid in fracture healing, primarily through the activation of the TGF-β/PI3K/Akt signaling pathway (48). Another study demonstrated that FN1 can effectively promote the differentiation as well as mineralization of osteoblasts by activating the WNT/β-catenin pathway (41), which can regulate bone-remodeling processes and play vital roles in the development of osteoporosis.

Follicle-stimulating hormone beta subunit (FSHB) is produced by the gonadotropic cells of the pituitary gland and plays a critical role in reproductive physiology as it can regulate gonadal growth, gametogenesis, and steroidogenesis (49). Our MR results showed that FSHB was positively associated with BMD, which was consistent with a previous study (50). This suggests that beyond its well-known role in reproduction, FSHB may also play a beneficial role in bone health. Moreover, studies in mouse embryonic fibroblasts have indicated that exogenous expression of FSHB could significantly increase BMP9-induced osteogenic differentiation (50). Additionally, FSHB was shown to be causally associated and directionally consistent with all osteoporosis-related traits measured by DXA. However, it has been reported that blocking FSHB can protect against bone loss in adult mice (51), suggesting that the effect of FSHB on BMD in adult mice may vary across different life stages, with potentially divergent outcomes in adult vs. young mice. These variations underscore the complexity of the role of FSHB in bone health and indicate the need for further research to fully understand its mechanisms and effects across different age groups.

The significant function of FSHB in the female reproductive system has been well-documented (52), which might explain the genetic association of FSHB with gynecological and obstetric disorders observed in Phewas analysis. This association could also account for the observed gender differences in the impact of FSHB on BMD. Phewas analysis showed that FSHB was predominantly associated with gynecologic and obstetric disorders, which may partially explain the gender differences in the genetic association between FSHB and HBMD. These findings could help to develop treatment strategies for different populations. BMI was identified as a mediating factor for the causal association between LRIG1, ACHE, FN1, FOXO1, and HBMD. These proteins were previously associated with lipid metabolism in previous studies (53, 54). Additionally, Phewas results analysis revealed a significant association of all these proteins with BMI. Consistent with a previous MR analysis (55), the PPI network in this study identified FN1, a key extracellular matrix glycoprotein, as the most crucial hub protein. Interestingly, among the drugs targeting the proteins identified, Donepezil and Rivastigmine, both ACHE inhibitors, were demonstrated to exert a protective influence on BMD (32), further validating the findings of our investigation.

LRIG1 and LRRC37A2 were novel proteins identified in our study, and potential explanations for their association with BMD were investigated. LRIG1, which fuses a leucine-rich repeat sequence and an immunoglobulin-like structural domain, exhibits significant tumor suppressor effects in cancer biology and is involved in fine regulatory processes in adult stem cells (SCs) (56). Previous studies have found that LRIG1 enhances the activity of the BMP signaling pathway (57). Basic experiments have strongly demonstrated that ectopic expression of LRIG1 in Lrig-null mouse embryonic fibroblasts (MRFs) effectively rescues the BMP signaling defect and restores its normal function (54). BMP has attracted attention for its ability to promote osteoblasts and chondrocyte differentiation, and its significant therapeutic efficacy in the field of bone defect treatment has also been widely verified (58). We hereby propose the hypothesis that BMP serves as a pivotal mediator in the potential genetic interplay between LRIG1 and osteoporosis. This hypothesis not only provides a new perspective for understanding the pathogenesis of osteoporosis but also signals that future studies may reveal new strategies for intervention and treatment of osteoporosis by regulating the LRIG1/BMP axis. To date, the research focus on LRRC37A2 has been predominantly directed toward the field of genetics, with its underlying biological mechanisms remaining largely underexplored. Previous genome-wide association studies (GWAS) have clearly revealed the causal role of LRRC37A2 in Parkinson's disease and breast cancer (59, 60), underscoring its immense potential as a biomarker and a promising drug target for both conditions. Notably, both breast cancer and Parkinson's disease patients exhibit an elevated risk for osteoporosis and bone loss (61, 62). Furthermore, postmenopausal women with osteoporosis are at a heightened risk of developing Parkinson's disease, a risk that can be mitigated, to a certain extent, through anti-osteoporosis treatments (63). In light of these intricate interconnections, it is plausible that LRRC37A2, through its involvement in the pathological processes of Parkinson's disease and breast cancer, may directly or indirectly impact bone metabolism, which may explain the genetic link between LRRC37A2 and BMD. Going forward, in-depth basic experimental studies are essential to elucidate the exact causal relationship between these two proteins and osteoporosis, which will provide a solid theoretical foundation for the development of effective therapeutic strategies against osteoporosis.

The key advantages of this study lie in its implementation of the latest proteomics data, stringent screening criteria, and multiple sensitive analyses, which collectively enhance the reliability of our conclusions. However, there are still several limitations exist. First, all the GWAS data was obtained exclusively from European populations. Given the genetic diversity among different ethnic groups, the applicability of these results to non-European populations remains to be determined. Second, number of instrumental variables from the corresponding protein dataset limited the ability to apply alternative MR methodologies, and conduct heterogeneity and pleiotropy tests. Thirdly, the influence of potential pleiotropy could not be completely excluded. However, all instrumental variables (IVs) were chosen based on strict criteria and demonstrated strong statistical power. Additionally, the inclusion of only cis SNPs in the MR analysis lends a high degree of biological plausibility to our findings.

## 5 Conclusion

Overall, our MR study has shed light on the significant roles played by certain proteins in the development of osteoporosis, revealing novel targets for potential therapeutic intervention. The study identified that proteins such as ACHE, HS6ST1, LRIG1, and LRRC37A2 have a negative association with BMD, whereas CELSR2, CPE, FN1, FOXO1, and FSHB have a positive association. Moreover, the associations of LRIG1 and LRRC37A2 with BMD have not been previously documented, marking an important contribution to the field. These discoveries underscore the importance of further investigations to delve into the mechanisms driving these associations and to explore their clinical relevance in osteoporosis treatment and management.

## Data Availability

The original contributions presented in the study are included in the article/Supplementary material, further inquiries can be directed to the corresponding author.
